# The prevention of pressure injuries in the positioning and mobilization of patients in the ICU: a good clinical practice document by the Italian Society of Anesthesia, Analgesia, Resuscitation and Intensive Care (SIAARTI)

**DOI:** 10.1186/s44158-022-00035-w

**Published:** 2022-01-31

**Authors:** Mariachiara Ippolito, Andrea Cortegiani, Gianni Biancofiore, Salvatore Caiffa, Antonio Corcione, Gian Domenico Giusti, Pasquale Iozzo, Alberto Lucchini, Paolo Pelosi, Gabriele Tomasoni, Antonino Giarratano

**Affiliations:** 1 Department of Surgical, Oncological and Oral Science (Di.Chir.On.S.), University of Palermo, Via del Vespro 129, 90127 Palermo, Italy; 2 Department of Anesthesia, Intensive Care and Emergency, Policlinico Paolo Giaccone, University of Palermo, Palermo, Italy; 3 UOC Anestesia e Rianimazione Trapianti Dipartimento di Patologia chirurgica, medica, molecolare e dell’Area Critica, Università di Pisa. Azienda Ospedaliera Universitaria Pisana, Pisa, Italy; 4 Intensive Care Respiratory Physiotherapy, Rehabilitation and Functional Education, San Martino Policlinico Hospital, IRCCS for Oncology and Neurosciences, 16132 Genoa, Italy; 5 Unit of Anaesthesia and intensive Care, Monaldi Hospital Naples, Naples, Italy; 6 School of Nursing, Azienda Ospedaliera Universitaria di Perugia, Perugia, Italy; 7 General Intensive Care Unit, Emergency Department - ASST Monza - San Gerardo Hospital, University of Milano-Bicocca, Via Pergolesi 33, Monza, MB Italy; 8 Department of Surgical Sciences and Integrated Diagnostics, University of Genoa, Genoa, Italy; 9 San Martino Policlinico Hospital, IRCCS for Oncology and Neurosciences, Genoa, Italy; 10 First Division of Anesthesiology and Critical Care Medicine, ASST Spedali Civili, Brescia, Italy

**Keywords:** Pressure injury, Pressure ulcers, Mobilization, Critical care

## Abstract

**Background:**

The aim of this document is to support clinical decision-making concerning positioning and mobilization of the critically ill patient in the early identification and resolution of risk factors (primary prevention) and in the early recognition of those most at risk (secondary prevention). The addresses of this document are physicians, nurses, physiotherapists, and other professionals involved in patient positioning in the intensive care unit (ICU).

**Methods:**

A consensus pathway was followed using the Nominal Focus Group and the Delphi Technique, integrating a phase of focused group discussion online and with a pre-coded guide to an individual phase. A multidisciplinary advisory board composed by nine experts on the topic contributed to both the phases of the process, to reach a consensus on four clinical questions positioning and mobilization of the critically ill patient.

**Results:**

The topics addressed by the clinical questions were the risks associated with obligatory positioning and therapeutic positions, the effective interventions in preventing pressure injuries, the appropriate instruments for screening for pressure injuries in the ICU, and the cost-effectiveness of preventive interventions relating to ICU positioning. A total of 27 statements addressing these clinical questions were produced by the panel. Among the statements, nine provided guidance on how to manage safely some specific patients’ positions, including the prone position; five suggested specific screening tools and patients’ factors to consider when assessing the individual risk of developing pressure injuries; five gave indications on mobilization and repositioning; and eight focused on the use of devices, such as positioners and preventive dressings.

**Conclusions:**

The statements may represent a practical guidance for a broad public of healthcare professionals involved in the management of critically ill patients.

**Supplementary Information:**

The online version contains supplementary material available at 10.1186/s44158-022-00035-w.

## Background

The stay in the intensive care unit (ICU) leads to multiple factors that limit mobility, such as hemodynamic instability, alteration of the sleep-wake rhythm, the use of invasive devices, the maintenance of forced positions for therapeutic purposes, and sedation to make mechanical ventilation more sustainable [1, 2]. In all the contexts of reduced patients’ mobility in which patients may suffer a reduction in mobility, the possible potential consequences of this functional limitation of this limitation must be acknowledged. A preventive and multidisciplinary approach to patient positioning and mobilization is essential to avoid complications, improve quality of life after discharge, and reduce health expenditure [3]. Indeed, pressure injuries are one of the main complications of positioning [4], and the prevalence of which in the ICU can be high. ICU admission frequently leaves negative long-term outcomes such as the so-called ICU-acquired weakness syndrome [5], as well as multiple neuropathies and myopathies [6]. The patient’s quality of life may also be negatively affected also from a cognitive, psychological, relational, and social point of view [7]. This is the rationale for the term “post-intensive care syndrome” [8]. Furthermore, the SARS-CoV-2 pandemic has highlighted the effect of prolongation of ICU admission times, together with the use of therapeutic positions, on long-term patient outcomes [9].

The aim of this document is to support clinical decision-making concerning positioning and mobilization of the critically ill patient in the early identification and resolution of risk factors (primary prevention) and in the early recognition of those most at risk (secondary prevention). The addressees of this document are physicians, nurses, physiotherapists, and other professionals involved in patient positioning in the ICU.

## Methods

The full version of the Italian document issued by the Italian Society of Anaesthesia, Analgesia, Resuscitation and Intensive Care (SIAARTI) was published in June 2021 and is freely available on the society’s website in Italian language [10].

For the purpose of this project, a consensus pathway was followed using the Nominal Focus Group—a technique of focused group discussion with experts that finds application within the Consensus Method—and the Delphi Technique for the “nominal” phase, integrating, therefore, a “real” phase of focused group discussion online and with a pre-coded guide to an individual phase—precisely, “nominal”. A multidisciplinary advisory board composed by nine experts on the topic (GB, SC, ACorc, AG, GG, PI, AL, PP, GT) contributed both to the nominal and to the real phases of the process.

### The process

The advisory board identified the core topics to be addressed with clinical questions and systematic searches. Four clinical questions regarding the prevention of pressure injuries in the positioning and mobilization of patients in the ICU (Table 1) were then formulated by a restricted working group, comprising four internal members of the Scientific Committee and two external reviewers.

**Table 1:** Clinical questions

• What are the risks associated with obligatory positioning and therapeutic positions? • What interventions are effective in preventing pressure injuries? • Which instrument is most appropriate for screening for pressure injuries in the ICU? • What cost-effectiveness evidence is available for preventive interventions relating to ICU positioning?	

A systematic review of the literature was conducted (by MI, AC) to provide the panel updated literature on the clinical questions. Literature reviews were conducted blind between the author and reviewers. Additional evidence was also gathered via a manual search and consultation with experts and Board members. The full methods of the systematic review are available in the Additional file 1. The clinical questions were then submitted to the advisory board, together with the results of the systematic searches to issue the good clinical practice statements based on the evidence and their clinical experience in the field. This took the form of an online submission of the material to be assessed, with a standardized Computer-Assisted Web Interview to collect the degree of agreement on the individual recommendations proposed. With the Delphi Technique, the degree of agreement (“Agreement”) of the experts on the good practices and the level of consensus (“Consensus”) among the experts on the assessment given to the good practices were found. For the final approval of the document, two rounds of Delphi were performed.

### Document structure

Each statement was reported with a classification of the level of evidence. Relevant references were inserted all along the document, with an indication of the category and level of evidence.

### Level of evidence

The classification of the level of evidence is presented in Table 2, and represented the strength and quality of the supporting study design. The level of evidence is also reported in Table 3 along each statement, and labeled as “Evidence”.

**Table 2:** Evidence classification

Category A: randomized controlled trials reporting comparative results for specific outcomes between different interventions.	
Level 1: meta-analysis of RCTs	
Level 2: Multiple RCTs of which a quantitative synthesis could not be conducted.	
Level 3: Single RCT.	
Category B: observational studies or trials without precise comparison groups, which may allow for an inference with respect to the relationship between interventions and observed outcomes.	
Level 1: non-randomized comparative studies (quasi-experimental, cohort studies, case-control).	
Level 2: non-comparative observational studies with measures of association (relative risk, correlation, sensitivity, and specificity).	
Level 3: non-comparative observational studies with descriptive measures (frequencies, proportions).	
Level 4: case reports and case series.

**Table 3:** Good practice summary table

1.1. Upper limb positioning			
Keep the upper limb in abduction at an angle of less than 90°, if necessary for therapeutic purposes			
Evidence: B3	Uncertainty: n.a.	Agreement: 4.7	Consent: Medium-Low
Maintain a trunk angle of between 15° and 30°			
Evidence: B1	Uncertainty: n.a.	Agreement: 4.7	Consent: Medium-High
1.2. Lower limb positioning			
The lower limb must not be hyperextended or spread more than 30° in supine and Trendelenburg positions			
Evidence: B2	Uncertainty: n.a.	Agreement: 4.4	Consent: Medium-low
1.3. Head positioning			
Carefully assess, in each individual case, the relationship between the expected benefit and possible risks of different degrees of head tilt in patients with severe head injury.			
Evidence: B1	Uncertainty: low	Agreement: 4.8	Consent: High
1.4. Prone position			
Prolonged maintenance of the prone position is associated with numerous complications, including serious complications. The expected benefit must outweigh the possible risks.			
Evidence: B1	Uncertainty: low	Agreement: 4.9	Consent: High
The prone position is used safely in patients with severe respiratory failure undergoing extracorporeal oxygenation.			
Evidence: B2	Uncertainty: high	Agreement: 4.3	Consent: Medium
The prone patient must be placed in the reverse Trendelenburg position with trunk tilt between 5° and 10°.			
Evidence: B2	Uncertainty: low	Agreement: 4.6	Consent: Medium-High
1.5. Supine position			
In the supine position, the trunk can be tilted between 10° and 28° without increased risk of pressure injuries. In the semi-supine position, the trunk can be tilted between 30° and 45° without increased risk of pressure injuries.			
Evidence: A2	Uncertainty: unclear	Agreement: 4.7	Consent: High
In the supine patient, keep the knees tilted between 5° and 10° and the heels elevated using a suspension device.			
Evidence: n.a.	Uncertainty: n.a.	Agreement: 4.3	Consent: Medium-High
2.1. Multidimensional risk assessment of positioning			
Integrate, into the patient’s care, an assessment of the risks associated with positioning that takes into account the patient’s individual risk factors, including age, body mass index, degree of mobility, perfusion status, blood glucose, and the existence of peripheral vasculopathy.			
Evidence: B2	Uncertainty: n.a.	Agreement: 4.6	Consent: Medium-High
2.2. Pressure Injury Risk Screening Tools			
The use of a validated screening scale for the risk of pressure injuries, sufficiently specific for the ICU context, allows for the early identification of those most at risk.			
Evidence: B1-B2	Uncertainty: high - unclear	Agreement: 4.4	Consent: High
The Braden scale corrected for albuminemia, known as the Braden scale (Alb), has sufficient sensitivity and specificity for use in the ICU and may be preferred to the uncorrected Braden scale.			
Evidence: B2	Uncertainty: low	Agreement: 4	Consent: Medium-Low
The Cubbin/Jackson scale has sufficient sensitivity and specificity for use in the ICU and may be preferred to the uncorrected Braden scale.			
Evidence: B2	Uncertainty: low	Agreement: 4.1	Consent: Medium
The CALCULATE scale has shown sufficient sensitivity and specificity for the use in the ICU and may be preferred to the uncorrected Braden scale.			
Evidence: B2	Uncertainty: low	Agreement: 4	Consent: Medium
3.1. Patient repositioning			
Adopt a patient repositioning protocol, customizing it based on the patient’s level of autonomy and availability of resources.			
Evidence: n.a.	Uncertainty: n.a.	Agreement: 4.8	Consent: High
Unconscious patient must be maintained in the lateral decubitus position. Any repositioning must be carried out by switching from one side to the other in accordance with the clinical condition.			
Evidence: A1-B1	Uncertainty: low	Agreement: 4.1	Consent: Medium-High
Adopting a patient repositioning feedback system, based on an electronic alert system or action protocol, reduces the incidence of pressure injuries.			
Evidence: A2	Uncertainty: low	Agreement: 4.4	Consent: High
The use of a motorized patient rotation and positioning device could reduce the incidence of pressure injuries and reduce staff fatigue, compared with manual repositioning.			
Evidence: B1	Uncertainty: high	Agreement: 4	Consent: Medium-High
3.2. Early mobilization			
ARDS patients on invasive mechanical ventilation for more than 24 h gain benefit from early mobilization.			
Evidence: A1	Uncertainty: high	Agreement: 5	Consent: Unanimity
4.1. Positioners			
Use a viscofluid head and neck positioning device whilst maintaining the lateral decubitus position.			
Evidence: B2	Uncertainty: high	Agreement: 5	Consent: Unanimity
Use a specific heel protector associated with passive mobilization.			
Evidence: A3	Uncertainty: low	Agreement: 5	Consent: Unanimity
4.2. Positioning surfaces			
Place the patient at risk of pressure injury on an air mattress.			
Evidence: A1	Uncertainty: low	Agreement: 4.9	Consent: High
Adopt two- or three-layer viscoelastic mattresses to prevent complications from immobility.			
Evidence: A2	Uncertainty: high	Agreement: 5	Consent: Unanimity
Use a visco-elastic foam mattress if it is not possible to reposition the patient at intervals of less than 4 h.			
Evidence: B1	Uncertainty: high	Agreement: 4.2	Consent: Medium-High
4.3. Preventive dressings			
Apply a multi-layered polyurethane foam preventive dressing with silicone to areas at risk of developing injuries, bony prominences, and areas subjected to pressure, rubbing, and shear forces.			
Evidence: A1	Uncertainty: low	Agreement: 4.8	Consent: High
4.4. Multi-intervention bundles			
Adopt a multidisciplinary protocol for proper positioning and prevention of pressure injuries.			
Evidence: B1	Uncertainty: high	Agreement: 4.9	Consent: High
Involve, according to specific protocols, external operators, experts in the treatment of complex wounds, and complications of immobility			
Evidence: B2	Uncertainty: high	Agreement: 4.7	Consent: High

Evidence was insufficient when studies analyzing specific interventions according to an established outcome were not collected, or the retrieved studies were not methodologically adequate. In few cases, indications have been formulated that were not supported by clear evidence but were considered valid, especially when they had been borrowed from previous guidelines, even though they lacked precise bibliographic references. In these cases, the level of evidence was classified as “undefined” (n.a.).

### Degree of uncertainty

Following a standardized assessment of the risk of bias in individual publications, the degree of uncertainty, labeled “Uncertainty” in the Table 3 of individual statements was reported as “high,” “unclear,” “low,” or “undefined (n.a.)”.

### Opinions

Statements that were not currently supported by evidence but based on the opinions of experts and board members were represented with O (“Opinion”). For them, neither the level of evidence nor the degree of uncertainty was reported.

### Degree of agreement

The degree of expert agreement on the good practice, labeled as “Agreement” in the Table 3, was represented as a central tendency index (mean value) of the scores assigned by the experts to the good practices using a rating scale of 1 to 5, where 1 represents the lowest level of agreement on the good practice and 5 represents the highest level, best supporting the statement.

### Consensus

The classification of the level of *Consensus* was based on the score dispersion index (standard deviations). It indicated how homogeneous or heterogeneous the panel’s opinion was, in a range from zero, the maximum homogeneity, to 1, the maximum dispersion. These values of standard deviations were then classified to form classes of consensus (with cutoffs to the indicated classes of 0; 0.5; 0.8; 0.9; 1). The level of consensus among experts on the rating given to good practice, labeled “Consensus” in the Table 3, was thus represented using a six-category classification: Unanimity, High, Medium-High, Medium-Low, and Low. The statements reaching a “Low” degree of consensus were excluded from the document.

## Results

**Fig. 1:**
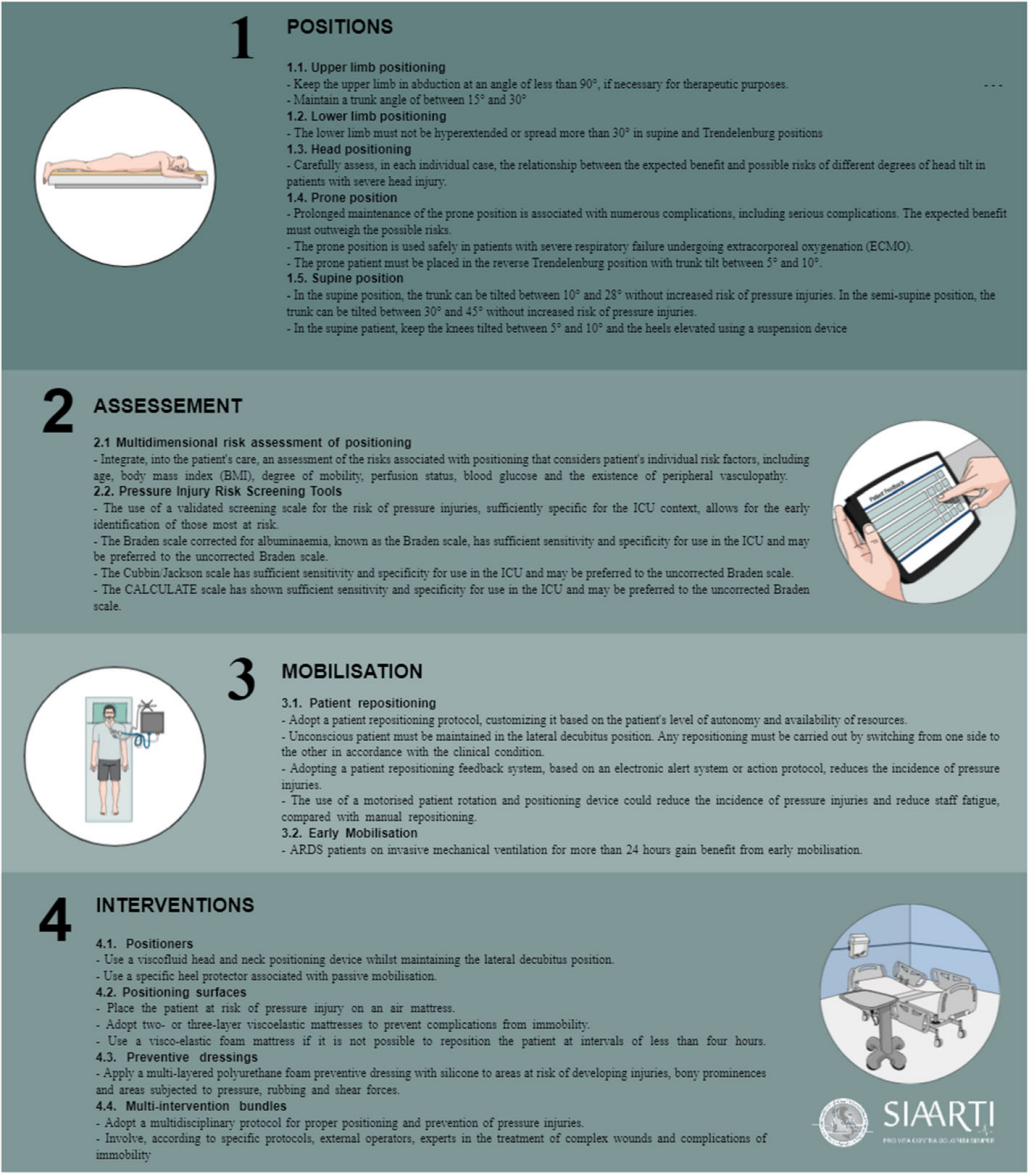
The figure represents a graphical summary of the experts' statements

During the process, only one statement reached a “Low” degree of consensus, being excluded from the document. At the final stage, a total of 27 statements were then produced and approved by the panel. The approved statements are presented in Table 3 and Fig. 1. The excluded statement is reported in Additional file 1.

*Statements*

### Upper limb positioning

#### Expert statement

*Keep the upper limb in abduction at an angle of less than 90°, if necessary for therapeutic purposes.*

#### Discussion

Arm abduction greater than or equal to 90° was associated with brachial neuropathy in three observational studies, although one RCT and one nonrandomized trial reported uncertain results [11]. A systematic review of case reports highlights the occurrence of upper limb neuropathy due to positioning under general anesthesia in various surgical disciplines, especially where limbs are in forced abduction. More damage has been reported to the lateral cutaneous nerve of the forearm [12].

#### Expert statement

*Maintain a trunk angle of between 15° and 30°*

#### Discussion

Trunk tilt between 15° and 30° prevents ulnar neuropathy, according to a non-randomized clinical trial [11].

### Lower limb positioning

#### Expert statement

*The lower limb must not be hyperextended or spread more than 30° in supine and Trendelenburg positions.*

#### Discussion

Hyperextension and divarication of the legs beyond 30° in the supine position promotes the onset of ischial neuropathy [11]. Hyperextension of the legs in the Trendelenburg position promotes the onset of femoral and obturator nerve neuropathy, according to an observational study [11].

### Head positioning

#### Expert statement

*Carefully assess, in each individual case, the relationship between the expected benefit and possible risks of different degrees of head tilt in patients with severe head injury.*

#### Discussion

According to a systematic review of three cross-over studies, therapeutic head positioning in the severe head injury patient is supported by very low-quality evidence. Currently, the balance of benefits and risks remains uncertain [13].

### Prone position

#### Rationale

Prolonged maintenance of the prone position for at least 12 h is associated with significantly reduced mortality in patients with acute respiratory distress syndrome (ARDS) [14]. Mortality is further reduced when prone positioning is combined with low current volume ventilation and when it is undertaken less than 48 h after the onset of the clinical condition [14–18].

The main adverse effects of prone positioning are the development of pressure injuries and endotracheal tube obstruction [15, 16]. The risk of developing pressure injuries in patients with ARDS is greater in the prone position than in the supine position [19]. Injuries can be caused by the effect of gravity on anatomical structures and the pressure created at points of contact between the body and underlying surfaces [20, 21]. The most frequent sites are the forehead, mandible, humerus, sternum, pelvic tuberosity, patella, and tibia [22].

#### Expert statement

*Prolonged maintenance of the prone position is associated with numerous complications, including serious complications. The expected benefit must outweigh the possible risks.*

#### Discussion

As described in a systematic review and reported in the Association of Perioperative Registered Nurses (AORN) guidelines, the prone position, used in surgical and resuscitative procedures, predisposes to increased intra-abdominal pressure, bleeding, abdominal and limb compartment syndrome, neuropathy, pressure injuries, cardiovascular decompensation, thrombosis and stroke, hepatic dysfunction, ocular damage, oropharyngeal oedema, airway maintenance dislocation, and gas embolism [23, 24].

#### Expert statement

*The prone position is used safely in patients with severe respiratory failure undergoing extracorporeal oxygenation (ECMO).*

#### Discussion

The safety of prone positioning in patients with severe respiratory failure treated with ECMO was investigated by a systematic review of seven observational studies that revealed limited complications [25]. The main complications were dislocation and bleeding at the level of the chest tube.

#### Expert statement

*The prone patient must be placed in the reverse Trendelenburg position with trunk tilt between 5° and 10°.*

#### Discussion

The risk of complications is increased when the prone patient is maintained in the Trendelenburg position [26]. Elevation of the head above the heart reduces venous congestion at the orbital and ocular levels. This reduces intraorbital and intraocular pressure. A systematic review and AORN guidelines suggest 5°–10° tilt in the prone patient in reverse Trendelenburg to reduce ocular complications [23–26].

### Supine position

#### Rationale

Prolonged supine maintenance exposes patients to the risk of developing pressure injuries in the occipital, scapular, vertebral, sacro-coccygeal, and calcaneal decubitus areas [22].

#### Expert statement

*In the supine position, the trunk can be tilted between 10° and 28° without an increased risk of pressure injuries. In the semi-supine position, the trunk can be tilted between 30° and 45° without increased risk of pressure injuries.*

#### Discussion

Different trunk tilts are recommended depending on the expected benefits with respect to the clinical condition and may expose differently to increased pressure and friction at different sites of injury. There were no significant differences, in terms of incidence of pressure injuries, between trunk tilt at 28° and 10° and between 45° and 30° [19].

#### Expert statement

*In the supine patient, keep the knees tilted between 5° and 10° and the heels elevated using a suspension device.*

#### Discussion

AORN guidelines recommend that the supine patient have the knees tilted 5–10° and the heels elevated via a suspension device [23]. Mobility is one of the three domains that affect the occurrence of pressure injuries, along with perfusion and skin condition [27].

### Multidimensional risk assessment of positioning

#### Expert statement

*Integrate, into the patient’s care, an assessment of the risks associated with positioning that considers patient's individual risk factors, including age, body mass index (BMI), degree of mobility, perfusion status, blood glucose, and the existence of peripheral vasculopathy.*

#### Discussion

In the ICU, age, degree of mobility, perfusion status, and use of vasopressors are risk factors for the development of pressure injuries, according to a systematic review of 17 studies [28]. Obesity, diabetes, age, vasculopathy, and low BMI have been associated with increased postoperative occurrence of peripheral neuropathy in several observational studies [11].

### Pressure Injury Risk Screening Tools

#### Expert statement

*The use of a validated screening scale for the risk of pressure injuries, sufficiently specific for the ICU context, allows for the early identification of those most at risk.*

#### Discussion

The Braden scale has been extensively validated in many care settings. According to a systematic review in 2013, it would also be the most widely tested scale in the critical care area [29]. Another systematic review noted the lack of evidence of effectiveness of the Braden scale in the ICU [30]. A subsequent meta-analysis of 11 studies and 10,044 participants concluded that the Braden scale has moderate predictive ability but is not sufficient to exclude an increased risk of pressure injuries in the ICU setting [31]. The accuracy of the Braden scale is significantly reduced in ventilated, dialyzed, inotropic, and surgical patients [32].

#### Expert statement

*The Braden scale corrected for albuminemia, known as the Braden scale, has sufficient sensitivity and specificity for use in the ICU and may be preferred to the uncorrected Braden scale.*

#### Discussion

The inclusion of albuminaemia in the Braden scale in the Braden version increased its specificity whilst maintaining good sensitivity [33].

#### Expert statement

*The Cubbin/Jackson scale has sufficient sensitivity and specificity for use in the ICU and may be preferred to the uncorrected Braden scale.*

#### Discussion

There are good alternatives to the Braden scale. The Cubbin-Jackson scale has similar predictive values to the Braden scale [29]. In a prospective observational study, the Cubbin/Jackson scale was indeed more specific than the Braden scale, whilst maintaining acceptable sensitivity [34]. However, the results may be affected by the small sample.

#### Expert statement

*The CALCULATE scale has shown sufficient sensitivity and specificity for use in the ICU and may be preferred to the uncorrected Braden scale.*

#### Discussion

The CALCULATE scale presented accuracy values comparable to the Braden [33]. The Norton scale reported accuracy values similar to the Braden scale, although it was affected by the small size of the samples [29]. The COMHON index and the Evaruci scale presented lower accuracy than the other aforementioned scales [35].

### Patient repositioning

#### Background

Patient repositioning is recommended in many guidelines in different ways, depending on the resources available [36].

#### Expert statement

*Adopt a patient repositioning protocol, customizing it based on the patient's level of autonomy and availability of resources.*

#### Discussion

The allocation of care resources and the determination of repositioning frequency must depend on a careful assessment of the patient’s degree of autonomy and activity and their ability to reposition themselves independently [37].

#### Expert statement

*Unconscious patient must be maintained in the lateral decubitus position. Any repositioning must be carried out by switching from one side to the other in accordance with the clinical condition.*

#### Discussion

According to a systematic review of 24 randomized and non-randomized studies, the evidence supporting repositioning of the unconscious patient in the lateral decubitus position rather than in other decubitus positions is not of adequate quality to draw firm conclusions [38]. However, according to a meta-analysis of 16 studies, the supine decubitus position results in reduced respiratory capacity in unconscious patients, whereas the lateral decubitus position would be characterized by greater safety [39].

#### Expert statement

*Adopting a patient repositioning feedback system, based on an electronic alert system or action protocol, reduces the incidence of pressure injuries.*

#### Discussion

Two RCTs conducted on 1534 patients studied repositioning every 2 h of the patient using either an electronic detection and notification system or a strategic intervention protocol. In both studies, the intervention resulted in a significant reduction in the incidence of injury [19].

#### Expert statement

*The use of a motorized patient rotation and positioning device could reduce the incidence of pressure injuries and reduce staff fatigue, compared with manual repositioning.*

#### Discussion

A prospective study of 717 patients investigated the use of the Prevalon® Motorised Rotation and Positioning System compared with manual positioning by caregivers. The device used consisted of two 30° inclined wedges with an attachment strap, a low friction slide sheet and a full body contact interface designed for microclimate management. The wedges and slip sheet are for individual use and the first layer against the patient’s skin is a disposable pad. This device, when compared with common lifts used, reduced the incidence of pressure injuries from 1.3 to 0%, with a significant 88% reduction in perceived exertion by staff [40].

### Early mobilization

#### Rationale

Active and passive mobilization interventions are aimed at recovering muscle tone, coordination, and range of joint movements and the performance of activities of daily living. Initial bedside interventions are followed by supine to bedside transfer exercises, from a sitting position to orthostaticism, the transition from bed to chair and walking exercises designed to improve postural stability, static and dynamic balance, and the resumption of walking with and without aids [1].

Early mobilization is even more important in ICU patients. Patients with acute lung injury present with symptoms known as Intensive Care Unit-Associated Weakness (ICUAW), which is accompanied by decline in physical function, as manifested by poor performance at the 6-min walk distance [41], up to 24 months after admission [17]. ICUAW has also been associated with increased mortality during hospitalization [42] and at 12 months after discharge [1, 43].

#### Expert statement

*ARDS patients on invasive mechanical ventilation for more than 24 h gain benefit from early mobilization.*

### Discussion

An early mobilization protocol for ARDS patients undergoing invasive mechanical ventilation for more than 24 h resulted in a reduction in total mechanical ventilation time, with reduced frequency of adverse events. There was no significant improvement in mortality or functional status of the patient at discharge [14, 44, 45]. In contrast, it appears that early mobilization does not significantly affect the quality of life of critically ill patients with severe brain damage [46].

Immobility causes pressure to be unloaded onto the areas of the body in contact with the positioning surface, which thus become sensitive areas for the onset of complications, first and foremost pressure injuries. Complications can be prevented by improving the quality of the interface between the body and the surface. This can be achieved by using suitable positioning surfaces and specific devices.

A device with physical characteristics such as to ensure an effect of wrapping and immersion of the part of the body concerned, thanks to the use of special materials capable of preserving the memory of the shape of the body part resting on them, increases the area on which the force given by the weight of the body is discharged, reducing tissue tension. At the same time, a material with viscoelastic properties tends not to change under the weight of the body, maintaining the correct alignment, thus reducing the tension that would result in increased pressure on the tissues [47, 48].

### Positioners

#### Expert statement

*Use a viscofluid head and neck positioning device whilst maintaining the lateral decubitus position.*

#### Discussion

A positioner made of a viscofluid material provided better maintenance of head and neck position and the lateral decubitus position [49]. In another comparison study with a historical cohort, head positioning of 127 patients on ECMO with the Z-Flo device significantly reduced the incidence of occipital injury [50]. In the study, the device was reshaped every 2 h by the operators, although this practice is not within the manufacturer’s guidance.

#### Expert statement

*Use a specific heel protector associated with passive mobilization.*

#### Discussion

The Prevalon® Heel Protector device combined with passive limb mobilization at each shift significantly reduced the incidence of heel injury in an RCT of 54 patients compared with the use of common cushions. In addition, a significant improvement in limb mobilization angle was found in prevention of contracture in plantar flexion [51].

### Positioning surfaces

#### Expert statement

*Place the patient at risk of pressure injury on an air mattress.*

#### Discussion

A meta-analysis of 65 studies concluded that there is a slight preventive effect of air mattresses against pressure injuries compared with conventional mattresses [52]. Based on the literature reviewed, best practices for the use of different anti-decubitus surface technologies cannot be outlined. It also remains for future research to understand whether it is appropriate to abandon the sheet when using technologically developed contact surfaces that minimize shear and pressure forces.

#### Expert statement

*Adopt two- or three-layer viscoelastic mattresses to prevent complications from immobility.*

#### Discussion

According to a recent systematic review, the evidence in favor of the use of anti-decubitus materials in intensive care is characterized by a high risk of bias, whilst the highest quality studies found no significant differences with common mattresses [53]. In some subsequent studies, the use of a viscoelastic pressure redistributing mattress has been shown to significantly reduce the incidence of pressure injuries [54]. No differences were observed between the use of two- or three-layer viscoelastic mattresses [19]. The use of alternating active pressure mattresses is of uncertain effectiveness [19].

#### Expert statement

*Use a visco-elastic foam mattress if it is not possible to reposition the patient at intervals of less than 4 h.*

#### Discussion

The use of a viscoelastic foam mattress with patient repositioning every 4 h had similar injury incidence compared with an air mattress with repositioning every 2 h [55].

### Preventive dressings

#### Expert statement

*Apply a multi-layered polyurethane foam preventive dressing with silicone to areas at risk of developing injuries, bony prominences, and areas subjected to pressure, rubbing, and shear forces.*

#### Discussion

The 2017 AORN guidelines recommend the use of preventative dressings on bony prominences or other areas subject to pressure, friction, and shear force [23]. Said preventive dressings significantly reduce the occurrence of pressure injuries [19, 56]. Although, until a few years ago, there was insufficient evidence to recommend a specific device [57], new studies have demonstrated the effectiveness of multi-layered polyurethane foam preventative dressings with silicone, which are also recommended by the NPIAP-EPUAP-PPPIA 20196 guidelines. The products tested in the literature are Mepilex® Border Sacrum, Mepilex® Border Heel, ALLEVYN® Gentle Border, and ALLEVYN® Life Sacrum [56]. The most studied sites are the heel and the sacrum [56, 58].

### Multi-intervention bundles

#### Expert statement

*Adopt a multidisciplinary protocol for proper positioning and prevention of pressure injuries. Involve, according to specific protocols, external operators, experts in the treatment of complex wounds, and complications of immobility*

#### Discussion

Implementation of a standardized pressure injury prevention and repositioning protocol could increase practitioner adherence to preventive practices [59]. An example in the literature is the Universal Pressure Ulcer Prevention (UPUP) Bundle. It suggests a process consisting of the application of emollients, complete skin assessment, distancing of the heels from the bed surface, early use of pressure redistribution surfaces, and repositioning of the patient. According to this protocol, the periodic presence in the intensive care unit of a nurse specializing in the treatment of complex wounds is also guaranteed [60]. Comparing the clinical activity before and after insertion of the UPUP Bundle, improved performance was observed, especially in patient repositioning and heel lift [60].

## Discussion

The main merit of the document is to provide a practical guidance, potentially useful for a broad range of healthcare professionals involved in the management of critically ill patients. Indeed, pressure injuries have a generalized impact on many aspects of life, can cause delay in the rehabilitation process or treatment of primary diseases, and contribute to a marked reduction in independence and autonomy of patients.

The 2019 updated NPIAP-EPUAP-PPPIA guidelines had previously defined the interventions for the prevention and treatment of pressure injuries [61]. In addition, the NSW Agency for Clinical Innovation (ACI) had produced guidelines for the prevention of pressure injuries in adult critical patients [62]. The American Society of Anesthesiologists (ASA) had also made recommendations for the prevention of perioperative peripheral neuropathy related to limb positioning [11].

There are also numerous observations in the literature regarding the quality of life of elderly people with pressure injuries, which can be analogously considered for patients admitted to the intensive care unit [7].

The preventive approach towards pressure injuries can have a significant economic return, and a reduction in health expenditure in terms of treatment of complications can be expected. Some studies have analyzed the economic impact of adopting specific interventions. Indeed, the cost-effectiveness of the use of some repositioning devices and preventive dressings have already been showed [40, 58]. Furthermore, although there is no clear evidence on this, we may assume a gain in terms of the quality-adjusted life years and disability-adjusted life years.

The document has limitations. Formal methods for guideline development were not applied. Thus, clinicians should continue referring to national and international guidelines on the topic. However, a rigorous consensus methodology was followed, and a systematic search of available evidence was performed. In this context, the document may provide a bedside support for healthcare professionals, as a complement to current guidelines.

## Conclusions

Critically ill patients are at risk of suffering from consequences of reduced mobility and forced positioning. A multidisciplinary panel of experts, including intensivists, critical care nurses, and physiotherapists provided a list of good clinical practice principles based on available evidence, by a structured method to analyze consensus. The statements may represent a practical guidance for a broad public of professionals involved in the management of critically ill patients.

## Supplementary Information

**Additional file 1.** Additional methods.

## Data Availability

The datasets used and/or analyzed during the current study are available from the corresponding author on reasonable request.

## Abbreviations

AORN: Association of Perioperative Registered Nurses
ARDS: Acute respiratory distress syndrome
BMI: Body mass index
ECMO: Extracorporeal membrane oxygenation
EUPAP: European Pressure Ulcer Advisory Panel
ICU: Intensive care unit
ICUAW: Intensive Care Unit-Associated Weakness
NPUAP: National Pressure Ulcer Advisory Panel
PPPIA: Pan Pacific Pressure Injury Alliance
RCT: Randomized controlled trial
SIAARTI: Italian Society of Anaesthesia, Analgesia and Intensive Care
UPUP: Universal Pressure Ulcer Prevention
