# Supplementary material for: The prevention of pressure injuries in the positioning and mobilization of patients in the ICU: a good clinical practice document by the Italian Society of Anesthesia, Analgesia, Resuscitation and Intensive Care (SIAARTI)
Source: J Anesth Analg Crit Care. 2022 Jan 31;2:7. doi: 10.1186/s44158-022-00035-w (PMC8802256; doi:10.1186/s44158-022-00035-w)
Supplement: Supplementary file 1 — Additional file 1. Additional methods. [file 44158_2022_35_MOESM1_ESM.docx]

Appendix to:

**Prevention of pressure injuries in the Intensive Care Unit: Expert consensus on positioning and mobilization by the Italian Society of Anaesthesia, Analgesia, Resuscitation and Intensive Care (SIAARTI)**

Mariachiara Ippolito^1,2^, Andrea Cortegiani^1,2*^, Gianni Biancofiore^3^, Salvatore Caiffa^4^, Antonio Corcione^5^, Giandomenico Giusti^6^, Pasquale Iozzo^2^, Alberto Lucchini^7^, Paolo Pelosi^8,9^, Gabriele Tomasoni^10^ and Antonino Giarratano^1,2^

^1^Department of Surgical, Oncological and Oral Science (Di.Chir.On.S.), University of Palermo, Italy

^2^Department of Anesthesia, Intensive Care and Emergency, Policlinico Paolo Giaccone, University of Palermo, Italy

^3^Department of Transplant Anesthesia and Critical Care, University School of Medicine, Pisa, Italy

^4^Intensive Care Respiratory Physiotherapy, Rehabilitation and Functional Education, San Martino Policlinico Hospital, IRCCS for Oncology and Neurosciences, 16132 Genoa, Italy

^5^Unit of Anaesthesia and intensive Care, Monaldi Hospital Naples, Italy, Naples, Italy

^6^School of Nursing, Azienda Ospedaliera Universitaria di Perugia, Perugia

^7^General Intensive Care Unit, Emergency Department - ASST Monza - San Gerardo Hospital, University of Milano-Bicocca, Via Pergolesi 33, Monza, MB, Italy

^8^Department of Surgical Sciences and Integrated Diagnostics, University of Genoa, Genoa, Italy.

^9^San Martino Policlinico Hospital, IRCCS for Oncology and Neurosciences, Genoa, Italy.

^10^First Division of Anesthesiology and Critical Care Medicine, ASST Spedali Civili, Brescia, Italy.

***Corresponding Author:** Andrea Cortegiani, MD, Prof., Department of Surgical, Oncological and Oral Science (Di.Chir.On.S.), University of Palermo, Italy. Department of Anaesthesia, Intensive Care and Emergency, Policlinico Paolo Giaccone, Palermo, Italy, Via del Vespro 129, 90127 Palermo, Italy. Email: andrea.cortegiani@unipa.it; Tel:+390916552730

**Content**

**Methods of preliminary systematic reviews performed**

**Research strategies**

**Excluded statement**

**Methods of preliminary systematic reviews performed**

The systematic reviews were performed according to the PRISMA Statement [1].

*Pressure injury (PI) prevention in the ICU*

A search string adapted for investigation in Embase, Medline and CINAHL was produced.

Studies, published from 1 January 2000 to 31 December 2020, conducted in populations older than 18 years of age admitted to intensive care units were included. Studies conducted in paediatric populations were excluded, even if in a paediatric intensive care setting. Studies conducted entirely in other settings were excluded. Studies conducted in different units, including ICU, were individually assessed for inclusion. Studies focusing on patients with ARDS, obesity and patients on invasive mechanical ventilation were investigated with particular interest.

Studies were included that addressed: the screening of PIs; the use of passive positioning devices and patient preventive dressings; the organisational arrangements of ICU staff with regard to the management of PIs; the economic analysis of PIs; and the impact of adopting preventive devices.

*Patient positioning in medical, surgical and intensive care settings*

A search was performed in MEDLINE and the Cochrane Library using a previously compiled string. The records found in the databases were analyzed in two steps: an initial screening by title and abstract and a more in-depth analysis on the selected articles.

The inclusion criteria were: populations older than 18 years; medical, surgical and intensive care settings; and reviews of observational and experimental studies.

Paediatric populations and studies performed in out-of-hospital or outpatient settings were excluded. Studies involving specific outcomes that were not transferable by analogy to the ICU context were excluded.

*Studies selection and data collection*

Articles from systematic reviews, meta-analyses, randomised controlled and uncontrolled trials and observational studies published in journals indexed in the databases reviewed were included. Studies of interest identified during the research or through references were also included manually. Conference abstracts, book chapters, narrative reviews, clinical case reports and case series were excluded.

The items extracted from the databases were collected and selected in two steps: a selection was made on titles and abstracts of all extracted items; potentially relevant articles were then divided into categories according to topic and examined in detail. Priority was given to systematic reviews already published. Studies already included in previous systematic reviews were not included. Distinctive features of some of these studies were highlighted in the data extraction of previous systematic reviews.

A table was created for each topic in question: risk factors and screening; passive positioning; aspects of pharmacoeconomics; organisational and management aspects. For each article included, the year of publication, number of participants, type and characteristics of the intervention, frequency or prevalence of LP and endpoints considered were indicated, if known. For studies that investigated the prevalence and risk factors of PIs in the ICU, prevalence, frequency and association with related risk factors were extracted. For studies assessing screening instruments, the name of the instrument, mode of administration, cut-off considered and diagnostic accuracy values were extracted. The main conclusions, where not expressible quantitatively and limitations of individual studies were also extracted.

*Qualitative assessment*

The quality of the included original studies was assessed using the following tools: RoB-2 for randomised controlled trials [2]; QUADAS-2 for diagnostic accuracy studies [3]; ROBINS-I for observational studies [4]; ROBIS for systematic reviews [5].

**Research strategies**

MEDLINE

The string has been adapted for searching in each database.

| **No.** | **String** | **Results** |
| --- | --- | --- |
| 1 | '"intensive care"[tiab] OR "critical care"[tiab] OR "critically ill patient"[tiab] OR  "critically ill"[tiab] OR "critically ill patient"[tiab] | 200686 |
| 2 | '"decubitus"[tiab] or "pressure ulcer"[tiab] or "pressure sore"[tiab] or "pressure  injur*"[tiab] or "pressure lesion*"[tiab]  or "decubitus ulce*"[tiab] or "bed sore"[tiab] or "bedsor*"[tiab] or "pressure- induced"[tiab] | 18172 |
| 3 | '"screening"[tiab] OR "multiple screening"[tiab] OR "prescreening"[tiab] OR  "project, screening"[tiab] OR "screening"[tiab] OR "screening method"[tiab] OR "screening procedure"[tiab] OR "screening program"[tiab] OR "screening programme"[tiab] OR "screening project"[tiab] OR "braden scale"[tiab] OR "braden scale for predicting pressure sore risk"[tiab] OR comhon[tiab] | 558310 |
| 4 | '"passive position"[tiab] OR "passive mobility"[tiab] OR microshift[tiab] OR  donut[tiab] OR "ring"[tiab] OR roho[tiab] OR "mölnlycke z-flo"[tiab] OR "dupaco proneview"[tiab] OR "head positioner"[tiab] OR "mattress"[tiab] OR surface[tiab] OR "neoprene"[tiab] OR "neoprene"[tiab] OR "neoprene rubber"[tiab] OR "polychloroprene"[tiab] OR "neoprene"[tiab] OR "reactive air"[tiab] OR "low air loss support"[tiab] OR "fluidised silicone"[tiab] OR "air filled"[tiab] OR "high specification foam"[tiab] OR "prophylactic"[tiab] or "preventative"[tiab] or "silicone foam"[tiab] or "multi-layered"[tiab] or "polyurethane"[tiab] or "hydrocolloid"[tiab] or "dressing"[tiab] or "Mepilex"[tiab] or "Allevyn"[tiab] or "OPSITE"[tiab] or "FLEXIFIT"[tiab] or "DuoDERM"[tiab] | 1375588 |
| 5 | 1 AND 2 AND 3 | 180 |
| 6 | 1 AND 2 AND 4 | 72 |
| 7 | 5 OR 6 | 242 |

**Excluded statement**

*Good practice: In ventilated patients, the risk of Ventilator-Associated Pneumonia (VAP) is not reduced by tilting the trunk at 45° compared with 10°.*

Uncertainty: n.a. Agreement: 3.7. Consent: Low

In patients on invasive ventilation, trunk tilt in the semi-supine position (trunk tilt between 30° and 60°) might be associated with a reduced risk of clinical syndrome suspected of ventilator-associated pneumonia (VAP) compared with the supine position (10° trunk tilt). However, the reported evidence has a high risk of bias. No clear benefits were observed in terms of mortality, duration of hospitalisation in ICU, duration of ventilation [6].

**REFERENCES**

1. Page M, McKenzie J, Bossuyt P, Boutron I, Hoffmann T, Mulrow C, et al. The PRISMA 2020 statement: an updated guideline for reporting systematic reviews. Bmj. 2021;372.

2. Sterne JAC, Savović J, Page MJ, Elbers RG, Blencowe NS, Boutron I, et al. RoB 2: A revised tool for assessing risk of bias in randomised trials. BMJ. 2019;366.

3. Whiting PF, Rutjes AWS, Westwood ME, Mallett S, Deeks JJ, Reitsma JB, et al. Quadas-2: A revised tool for the quality assessment of diagnostic accuracy studies. Ann Intern Med. 2011;155:529–36.

4. Sterne JA, Hernán MA, Reeves BC, Savović J, Berkman ND, Viswanathan M, et al. ROBINS-I: A tool for assessing risk of bias in non-randomised studies of interventions. BMJ. 2016;355.

5. Whiting P, Savović J, Higgins JPT, Caldwell DM, Reeves BC, Shea B, et al. ROBIS: A new tool to assess risk of bias in systematic reviews was developed. J Clin Epidemiol. 2016;69:225–34.

6. Wang L, Li X, Yang Z, Tang X, Yuan Q, Deng L, et al. Semi-recumbent position versus supine position for the prevention of ventilator-associated pneumonia in adults requiring mechanical ventilation. Cochrane Database Syst Rev. 2016;2016.
